# Colitis promotes neuronal differentiation of Sox2+ and PLP1+ enteric cells

**DOI:** 10.1038/s41598-017-02890-y

**Published:** 2017-05-31

**Authors:** Jaime Belkind-Gerson, Hannah K. Graham, Justin Reynolds, Ryo Hotta, Nandor Nagy, Lily Cheng, Michal Kamionek, Hai Ning Shi, Carol M. Aherne, Allan M. Goldstein

**Affiliations:** 1 Neurogastroenterology Program, Digestive Health Institute, Children’s Hospital Colorado University of Colorado, Aurora, USA; 2 Pediatric Surgery, Massachusetts General Hospital, Harvard Medical School, Boston, MA USA; 3 Center for Neurointestinal Health, Massachusetts General Hospital, Harvard Medical School, Boston, MA USA; 4 Pathology department, Carolinas Healthcare System, Charlotte, NC USA; 5 Department of Anesthesiology, Mucosal Inflammation Program, University of Colorado School of Medicine, Aurora, USA

## Abstract

Mechanisms mediating adult enteric neurogenesis are largely unknown. Using inflammation-associated neurogenesis models and a transgenic approach, we aimed to understand the cell-source for new neurons in infectious and inflammatory colitis. Dextran sodium sulfate (DSS) and Citrobacter rodentium colitis (CC) was induced in adult mice and colonic neurons were quantified. Sox2GFP and PLP1GFP mice confirmed the cell-type specificity of these markers. Sox2CreER:YFP and PLP1creER:tdT mice were used to determine the fate of these cells after colitis. Sox2 expression was investigated in colonic neurons of human patients with *Clostridium difficile* or ulcerative colitis. Both DSS and CC led to increased colonic neurons. Following colitis in adult Sox2CreER:YFP mice, YFP initially expressed predominantly by glia becomes expressed by neurons following colitis, without observable DNA replication. Similarly in PLP1CreER:tdT mice, PLP1 cells that co-express S100b but not RET also give rise to neurons following colitis. In human colitis, Sox2-expressing neurons increase from 1–2% to an average 14% in colitis. The new neurons predominantly express calretinin, thus appear to be excitatory. These results suggest that colitis promotes rapid enteric neurogenesis in adult mice and humans through differentiation of Sox2- and PLP1-expressing cells, which represent enteric glia and/or neural progenitors. Further defining neurogenesis will improve understanding and treatment of injury-associated intestinal motility/sensory disorders.

## Introduction

Injury to the enteric nervous system (ENS) occurs throughout life due to inflammation, metabolic disarray, infection, dysbiosis, toxins, surgery and aging. These insults can lead to disordered gastrointestinal motor and sensory function in conditions such as esophageal achalasia, gastroparesis, irritable bowel syndrome, and slow transit constipation^1, 2^. In the central nervous system (CNS), constitutive neurogenesis occurs at a low rate and neuronal injury triggers an accelerated birth of new neurons to replace those that are damaged^3^. In contrast, constitutive neurogenesis has not been identified in the ENS beyond the first several months of postnatal life^4^. Even following injury, enteric neurogenesis has not been consistently observed. Laranjeira *et al.* found that chemical denervation of the gut leads to the birth of new neurons^5^, while others have failed to identify neurogenesis using a wide variety of injury models^6^. We recently demonstrated robust neurogenesis following induction of chemical colitis in adult mice^7^ and showed that this response depends on serotonin (5-HT) signaling through its 5-HT_4_ receptor^7^. In this model, proteins normally expressed by enteric glia, and not enteric neurons (Sox2, Nestin, CD49b), become expressed in a subset of neurons^7^, suggesting that inflammation induces neurogenesis from a population of cells expressing these markers. The nature of this neurogenic cell type, however, is unknown.

The possibility that enteric glia can give rise to enteric neurons in specific situations has been proposed by others^8^ using both *in vitro* ^6^ and *in vivo* ^5^ models. However, *in vivo* neurogenesis has only been described using either a prolonged course of a 5-HT_4_ agonist or after an aggressive detergent-mediated denervation model using trans-serosal benzalkonium chloride (BAC)^4^. It is not clear that neurogenesis occurs in association with common forms of intestinal injury^6^. If glial cells are capable of neurogenesis *in vivo*, the immunophenotype of those glial cells remains unknown. Indeed, glial cells are known to be plastic^9^ and have previously been shown to undergo neuronal transdifferentiation in the CNS^10, 11^. Sox2, for example, is able to reprogram resident astrocytes into proliferative neuroblasts in the adult mouse brain^12^. This same plasticity may exist in the intestinal tract, but has not been consistently demonstrated. Here we present evidence that enteric cells expressing Sox2 and PLP1, markers that label enteric glia in the adult gut, undergo neurogenesis in response to colitis.

## Materials and Method

### Mice

All methods were performed in accordance with the relevant guidelines and regulations after obtaining Massachusetts General Hospital IACUC approval. C57BL/6 wild-type mice were used as controls. Sox2GFP and tamoxifen-inducible SOX2:iCreERT2:R26ReYFP mice (SOX2CreER:YFP) were generously provided by Konrad Hochedlinger^13^. PLP1GFP tissue was generously provided by Wendy Macklin^14^. Tamoxifen-inducible PLP1:iCreERT2 were mated to tdTomato reporter mice (both from The Jackson Laboratory) to yield PLP1CreER:tdT offspring (Table 1).

**Table 1:** Mouse models used in the current work.

nomenclature	breeding	Source	ref	Image
Sox2-GFP	Heterozygote × Wild-type	Konrad Hoechedlinger, MGH	13	Fig. 2
Sox2CreER:YFP	Sox2CreER male × ROSA26-lox-STOP-lox(lsl)-EYFP female	Konrad Hoechedlinger, MGH	13	Figs 2, 3
PLP1-GFP	Heterozygote × Wild-type	Wendy Macklin University of Colorado, Denver	14	Fig. 4
PLP1CreER:tdT	PLP1CreER male × ROSA26-lox-STOP-lox tdT female	The Jackson Laboratory (B6.Cg-Tg(Plp1-cre/ERT)3Pop/J)	15	Figs 4–6
Ret-CFP	Heterozygote × Wild-type	Meenakshi Rao, Columbia Univ.	16	Fig. 7

### Dextran sulfate sodium (DSS) colitis

Colitis was induced by adding 3% dextran sulfate sodium to (DSS; MP Biomedicals, Santa Ana, CA Reagent Grade Cat. #160110, mol weight 40,000) the drinking water of 4 month-old C57BL/6 mice (Jackson Laboratory, Bar Harbor, ME) for 7 days. Mice were sacrificed 48–72 hours after colitis and the colon examined by immunohistochemistry. The experiment was done using 4 colitis mice and 4 age-matched controls and again repeated with 4 new colitic mice and 4 age-matched controls. The mice were aged 11–12 weeks at the start of DSS and sacrificed on day 7 after 6 days of DSS and 1 day of water.

### Citrobacter rodentium colitis

*C. rodentium* (strain DBS100, ATCC) was grown in Luria broth (LB), resuspended in PBS (0.5 ml/mouse, 5 × 10^8^ CFU of *C. rodentium*) and fed to 4 month-old C57BL/6 mice^17^. Mice were sacrificed 10 days later and colon collected for immunohistochemistry. *C. rodentium* colitis was induced in 8 mice aged 4 months old, while 6 age-matched control mice were used.

### Cell quantification after colitis

The colon was removed from colitis and control mice immediately after sacrifice and dissected to obtain laminar preparations of the wall of the colon (longitudinal muscularis, myenteric plexus prep or LMMP)^7^ as well as paraffin sections of the colon, which were cut longitudinally. Laminar preparations were fixed as described above, processed for immunofluorescence, and examined as whole mounts. Paraffin sections were also stained using immunofluorescence and photographed. The densities of neurons, identified in the laminar preparations as described above, were determined and expressed as a function of tissue area (mm^2^) and also as average number of neurons per ganglia following colitis. Individual neuronal density were defined as the sum of neurons counted, using a 20X objective, in at least 20 contiguous, non-overlapping fields. For sections, the sum of neurons counted, using a 40X objective, in at least 20 contiguous, non-overlapping fields were quantified. For cell quantification, imageJ was used and manually verified. The densities of neurons in the colon of mice aged 4 months old were compared to those measured in the WT controls. For DSS colitis 4 colitic mice and 4 age-matched wild type controls that were not given DSS and had no colitis were studied. For *C. rodentium* colitis mice, 8 mice 4 months old and 6 age-matched control mice were used. These were used to determine the average number of neurons per mm^2^ of tissue. All cell counting was performed by a blinded reader who counted from de-identified images that included only the myenteric plexus and not the mucosa.

To make sure that the number of neurons per mm^2^ of tissue was not altered by tissue contraction due to colitis, we repeated the experiment (n = 4 DSS and 4 controls). Gender, age and weight-matched C57BL/6 mice were exposed to 3% DSS (36,000–50,000 MW; MP Biomedicals, Solon, OH) in their drinking water or water alone. Following 7 days whole colon was harvested by blunt dissection and fixed in 10% buffered-formalin (Sigma Aldrich, St. Louis, MI) followed by embedding in paraffin. Tissues were sectioned, deparaffinized and re-hydrated through descending series of ethanol/water baths. Antigen retrieval was performed in citrate buffer (Vector Labs) and blocked with 1% BSA in PBS. Tissues were incubated with primary antibody Anti-Anna-1 (1:10,000) overnight at 4 °C. Slides were washed in PBS and incubated with secondary antibody (goat anti-human Alexa Fluor 594) (1:1,000). Following washing in PBS slides were incubated with DAPI nuclear counterstain reagent (Life Technologies, NY) for 10minutes. Tissues were mounted in ProLong® Gold Antifade reagent (Life Technologies, NY). The primary antibody was omitted as negative control. No specific signal was observed in the negative control. Images were acquired using an Olympus BX51 with an Olympus DP72 camera and CellSens imaging software (V1.6). Images were also obtained with a Nikon A1 confocal microscope equipped with galvo and resonant scanners for high resolution scanning.

### Cell proliferation *in vivo*

Intraperitoneal injection with 50 mg/kg EdU (5-ethynyl-2-deoxyuridine) was performed every 24 hours during DSS administration for a total of 7 doses. The distal colon was removed, fixed in 10% formalin, and EdU incorporation detected using the ClickiT EdU Imaging Kit (Life Technologies) per manufacturer’s instructions. EdU incorporation was assessed both in paraffin sections as well as in whole-mount LMMP preparations.

### Clinical and histologic scoring of colitis

Mice were assessed for colitis clinically by quantifying weight loss and identifying blood (gross or microscopic) in stools. A blinded pathologist scored the severity of colitis on H&E-stained slides using a grading scale modified from Mizoguchi E. *et al*.^18^ The mean colitis score (range 0–6) was calculated based on twenty random high-power fields per colon.

### Immunohistochemistry

Immunohistochemistry was performed as described^19^. The following primary antibodies were used: Human anti-Anna-1 (Hu) positive serum (1:16,000, generous gift from Dr. Vanda Lennon; Mayo Clinic, Rochester MN;), goat anti-Sox2 (1:50; R&D Systems, Minneapolis, MN, Cat # AF2018), mouse anti-Tuj1 (1:100; Covance, Dedham, MA Cat # MMS-435P), goat anti-GFAP (1:500; Abcam. Cat # ab53554), Rabbit anti-PGP 9.5 (1:200; Abcam. Cat # ab27053), Rabbit anti-RET (1:100; Alomone labs, Israel, Cat #: ANT-025), goat anti-GFP (1:200; Rockland Cat # 600-101-215), Rabbit anti-S100b (1:100; Neomarkers, Cat # RB-044-A). Rabbit anti-Calretinin (Invitrogen 18-0211) Anti-Rabbit nNOS (Santa Cruz Cat# SC648), Anti-rabbit synaptophysin (Abcam Cat# ab1469). Secondary antibodies included goat anti-mouse IgG Alexa Fluor 546, goat anti-rabbit Alexa Fluor 488, donkey anti-goat Alexa Fluor 488, donkey anti-goat Alexa Fluor 546, donkey anti-mouse Alexa Fluor 546 and goat anti-human Alexa Fluor 594, all from Invitrogen (Carlsbad, CA). Cell nuclei were stained with DAPI (Vector Labs, Burlingame, CA).

### Preparation of neurosphere-like bodies (NLBs)

All animal experiments were performed with institutional approval. C57BL/6 mice were sacrificed on postnatal day 7–21 (P7–21) and the colon dissociated with dispase (250 μg/ml; StemCell Technologies, Vancouver, Canada) and collagenase XI (1mg/ml; Sigma-Aldrich, St. Louis, MO) at 37 °C for 1 hour with gentle pipetting. The cell suspension was passed through a 40 μm cell strainer and cultured at a density of 50,000 cells/mL in NeuroCult proliferation medium (StemCell Technologies) containing 20 ng/ml epidermal growth factor (EGF; StemCell Technologies) and 10 ng/ml basic fibroblast growth factor (bFGF; StemCell Technologies) for 7–10 days on fibronectin-coated dishes to form neurosphere-like bodies (NLBs). NLBs were dissociated with Accutase (StemCell Technologies) at 37 °C for 30 minutes with gentle pipetting. The cell suspension was passed through a 40 μm cell strainer and plated on fibronectin-coated glass-bottom chamber slides (Biomedical Technologies, Ward Hill, MA). Cells were cultured for 7 days in neural differentiation medium (NeuroCult; StemCell Technologies), then were processed for immunohistochemistry. For neurosphere experiments using CreER-derived tissue, Tamoxifen (2mg) diluted in corn oil, was given via intraperitoneal (i.p.) injection to the mice the day prior to sacrifice.

### Catenary LMMP cultures

Colonic longitudinal muscle-myenteric plexus (LMMP) was prepared from 6mo PLP1CreER:tdT mice and cultured ex vivo for 3–6 days. The mouse received one tamoxifen dose (2mg) by i.p. injection 24 hours before sacrifice. The Dissected LMMP tissue was suspended across a triangular shaped orifice that had been cut into pieces of filter paper. The hanging tissue obtains nutrition by diffusion and survives several days in culture as has been described previously^20^. The tissue was then fixed in 4% paraformaldehyde.

### Cell proliferation and apoptosis *in vitro*

To detect cell proliferation, dissociated NLBs were cultured in the presence of 10 μM EdU, for 24 hours. EdU incorporation was detected using the Click-iT EdU Imaging Kit (Life Technologies; Carlsbad, CA).

### Human tissue

All methods for humans were performed in accordance with the relevant guidelines and regulations. After obtaining Massachusetts General Hospital Institutional Review Board approval, de-identified tissue from patients who underwent colectomy for *Clostridium dificile* colitis or ulcerative colitis was obtained from the pathology department of the Carolinas Health System. Controls consisted of healthy margins of colon resected for cancer.

### Statistical analysis

Data are expressed as means ± SD. Unpaired t-tests were used to evaluate the statistical significance between two groups. When more then two groups were compared, means were statistically compared by one-way ANOVA with Tukey post hoc multiple-comparison tests using the Prism computer software program. Statistical significance was considered at a cutoff of p < 0.05.

## Results

### Chemical and infectious colitis induce early colonic neurogenesis

**Figure 1:**
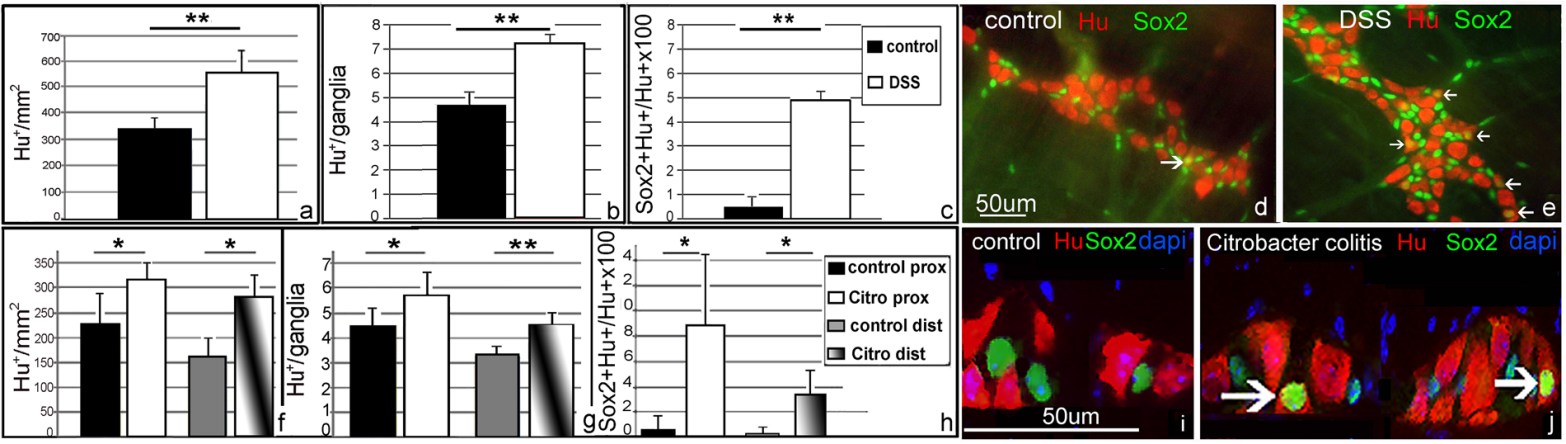
Chemical and infectious colitis both promote enteric neurogenesis. DSS colitis (n = 8) and *Citrobacter rodentium* colitis (n = 8) were induced in 4 month-old mice and the colons processed for immunohistochemistry using Hu antibody to label enteric neurons. Significantly more neurons are present throughout the colon in both DSS (**a**,**b**) and *Citrobacter rodentium* (**f**,**g**) models. This increased neuronal density is associated with an increase in the proportion of Hu+ neurons that label with Sox2 (c-e and h-j, arrows point to Hu + Sox2+ neurons in (**d**,**e** and **j**). *p < 0.05, **p < 0.01. (**d** and **e**) were taken at 200x, (**i** and **j**) at 400x).

Acute colitis was induced with DSS in adult 4 month-old mice as previously described^7^. At day 10, 3 days after completing DSS, total colonic neuronal numbers were substantially increased by 59%, from 330 ± 48 to 553 ± 95 per mm^2^ (Fig. 1a, representative images in d,e). To confirm this observation in a second model of colitis, infectious colitis was induced by delivering *Citrobacter rodentium* enterally to 4 month-old C57BL/6 mice. *C. rodentium* is a gram negative organism that colonizes the distal colon and causes pathological changes including transmissible colonic hyperplasia, goblet cell depletion, and mucosal erosion^17, 21, 22^. Following *C. rodentium* infection, neuronal density was significantly increased by 27% and 43% in the proximal and distal colon, respectively, as shown by the density of Hu+ enteric neurons (Fig. 1f). Sox2 normally labels the majority of enteric glia in the postnatal gut^23^. When we labeled glial cells with Sox2 to observe any inflammation-associated changes in enteric glia, we noted that in control colon almost no neurons in the proximal and distal colon (0.6% and 0.3% of Hu+ enteric neurons, respectively), co-express Sox2. However, following *C. rodentium* induced colitis, the number of Sox2+ neurons increased significantly both proximally (8.9%) and distally (3.4%) (Fig. 1h–j). In a similar fashion, Sox2+ neurons were significantly increased after DSS colitis (0.5 in control and 4.9% after DSS) (Fig. 1c).

To make sure that the number of neurons per mm^2^ of tissue was not altered by tissue contraction due to colitis, we also quantified the average number of neurons per ganglion following DSS and *C. rodentium* colitis. The results showed a 35% increase in number of neurons per ganglion following DSS colitis (control: 4.7 ± 0.51; DSS: 7.23 ± 1 0.38; p < 0.001; Fig. 1b) and in *C. rodentium* colitis a 23.5% increase in number of neurons per ganglion in the proximal colon and 28.9% increase in the distal colon (proximal control: 4.5 ± 0.7; proximal *C. rodentium*: 5.8 ± 0.7, p < 0.05; and distal control: 3.2 ± 0.3; distal *C. rodentium*: 4.6 ± 0.4, p < 0.01; Fig. 1g).

### Identifying the neurogenic cell source

**Figure 2:**
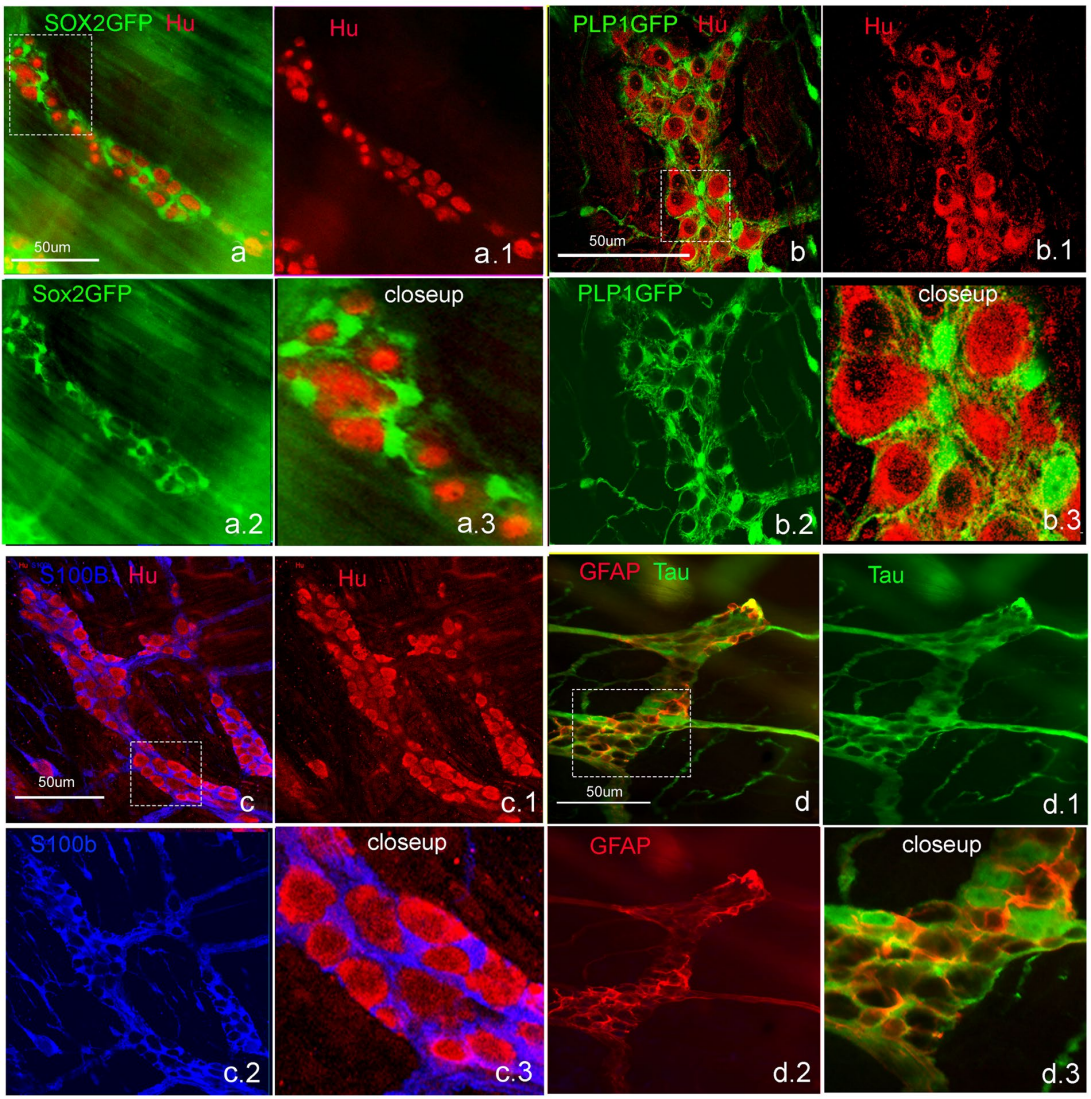
Sox2, PLP1 and S100b label enteric glia. Sox2GFP (**a**) labels most enteric glial cells in the adult mouse colon. Similarly, PLP1GFP (**b**) also labels most enteric glial cells. S100b is a third marker labeling most glial cells (**c**). GFAP, however only labels a fraction of enteric glia (**d**,**d.3** close up of boxed area in **d**). (All Figures were taken at 200x, except closeup maginfications).

**Figure 3:**
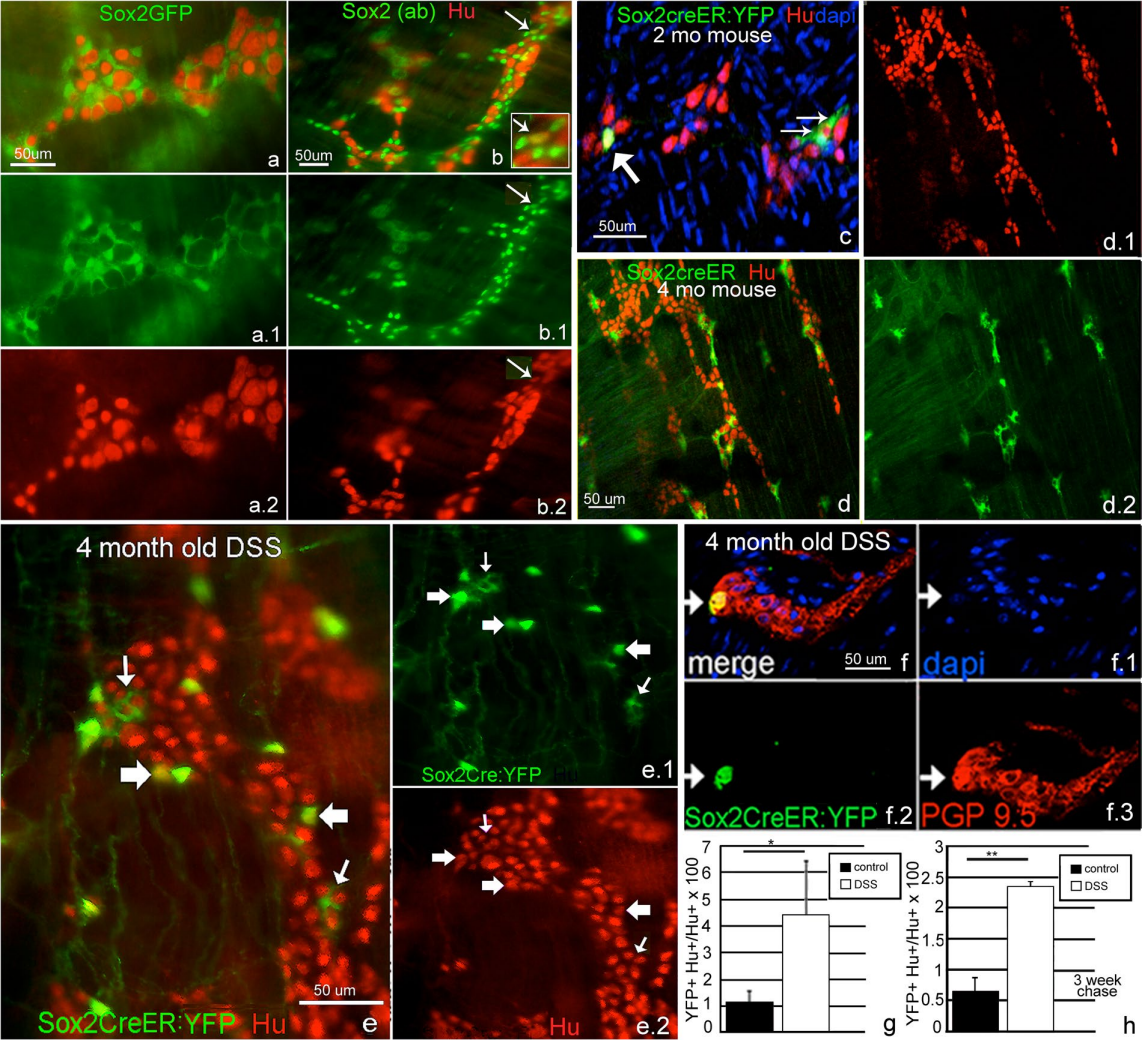
Colitis induces differentiation of Sox2+ glial cells to enteric neurons. Sox2 is normally expressed in the adult colon in glia both in the SOX2GFP mouse (**a**) as well as using anti-Sox2 antibodies (Sox2(ab)), as shown in (**b**). After 4 months of age, only occasional neurons express Sox2 protein (**b**, arrow). At 2 months of age, 3.5% of Hu+ colonic neurons express YFP (**c**, thick arrow), but in the Sox2CreER as in the other mouse models, these are rare by 4 months, when Sox2 expression is primarily limited to glia (**d**). After tamoxifen, DSS was given to 4 month-old mice for 7 days, followed by sacrifice on day 10. Treatment with DSS leads to a significant increase in YFP-expressing Hu+ neurons (**e**), thick arrows; thin arrows point to YFP+ glial cells). YFP+ neurons are also positive for PGP9.5 (**f**). In 4 month-old mice, the proportion of Hu+ neurons that express YFP increases from 1.1 ± 0.6% in controls to 4.4% ± 1.9% after colitis (**g**). A similar increase is observed when tamoxifen is administered 3 weeks before DSS, ensuring that residual tamoxifen is no longer present, 0.7% ± 0.2 in control and 2.4% ± 0.1 after DSS (**h**), (**p < 0.01) n = 4. (**a**,**c**,**e** and **f**) were taken at 200x, (**b** and **d**) at 100x).

Based on the hypothesis that the neurogenic cell may have a glial immunophenotype, we used Sox2GFP and PLP1GFP models. Both markers have widespread glial expression (Fig. 2a,b), very similar to S100b, which is expressed broadly by mature enteric glia (Fig. 2c), whereas GFAP is expressed only in a subset of these cells (Fig. 3d).

### Sox-2 expressing cells give rise to neurons following colitis

Given the increase in Sox2-expressing neurons following colitis in adult animals (Fig. 1f–h), we hypothesized that Sox2-expressing cells give rise to neurons. We performed cell-fate studies using a Sox2 reporter line. Undifferentiated enteric neural crest cells in the developing ENS express Sox2 before becoming restricted to enteric glia following their differentiation^23^. Co-expression of Sox2 with S100b (Fig. 2a,c) supports glial expression of this transcription factor. In Sox2GFP adult mice, GFP is rarely expressed by enteric neurons (Fig. 3a). Sox2 immunofluorescence confirms this observation (Fig. 3b, arrow). To test whether Sox2+ cells give rise to new neurons, a genetic lineage tracing approach was employed using a tamoxifen-inducible Cre (CreER-T2) inserted in the endogenous Sox2 locus with additional targeting of a ROSA26-lox-STOP-lox (lsl)-EYFP reporter. The inducibility and specificity of this system has been previously validated^13^. Sox2-CreER:ROSA26-lsl-EYFP mice (Sox2CreER:YFP) not given tamoxifen, and those treated with vehicle (corn oil) alone, exhibit very rare, spontaneous labeling events in the stomach^13^ and none in the colon.

To label Sox2-expressing cells and their progeny *in vivo*, tamoxifen was administered intraperitoneally as a single dose into Sox2CreER:YFP animals. In 2 month-old mice, 3.5 ± 2.2% of Hu+ neurons express YFP (Fig. 3c) n = 4, likely due to ongoing postnatal neurogenesis from Sox2-expressing precursors at this young age. By 4 months of age, however, YFP expression in Hu+ neurons is infrequently observed in normal mice, with only 0.7 ± 1.1% showing co-expression (Fig. 3d) n = 4. To avoid the potential early postnatal neurogenesis we only used mice 4 months or older. We added DSS to the drinking water of 4 month-old mice for 7 days, starting 1 day after tamoxifen administration, and analyzed the colon on day 10. This resulted in a dramatic rise in the number of YFP+Hu+ enteric neurons (Fig. 3e). These YFP+ neurons also co-express PGP9.5, a pan-neuronal marker in the gut (Fig. 3f). In 4 month-old mice, YFP + Hu+ double-expressing neurons rose in frequency from 1.1 ± 0.6% of all Hu+ cells in controls to 4.4% ± 1.9% following DSS colitis (Fig. 3g; p < 0.05), suggesting that colitis causes Sox2+ cells to give rise to new neurons. Since DSS was started one day following tamoxifen administration, residual circulating tamoxifen could still be inducing ongoing Cre recombination. To exclude this, the experiment was repeated by feeding DSS 3 weeks after tamoxifen administration. The results were similar, with colitis contributing to an increase in the percentage of YFP+ neurons from 0.7% ± 0.2 to 2.4% ± 0.1 in control and DSS-treated mice, respectively (Fig. 3h; p < 0.01). These results suggest that Sox2-expressing cells give rise to new neurons in response to inflammation.

### Sox2+ cells give rise to neurons without undergoing cell division

**Figure 4:**
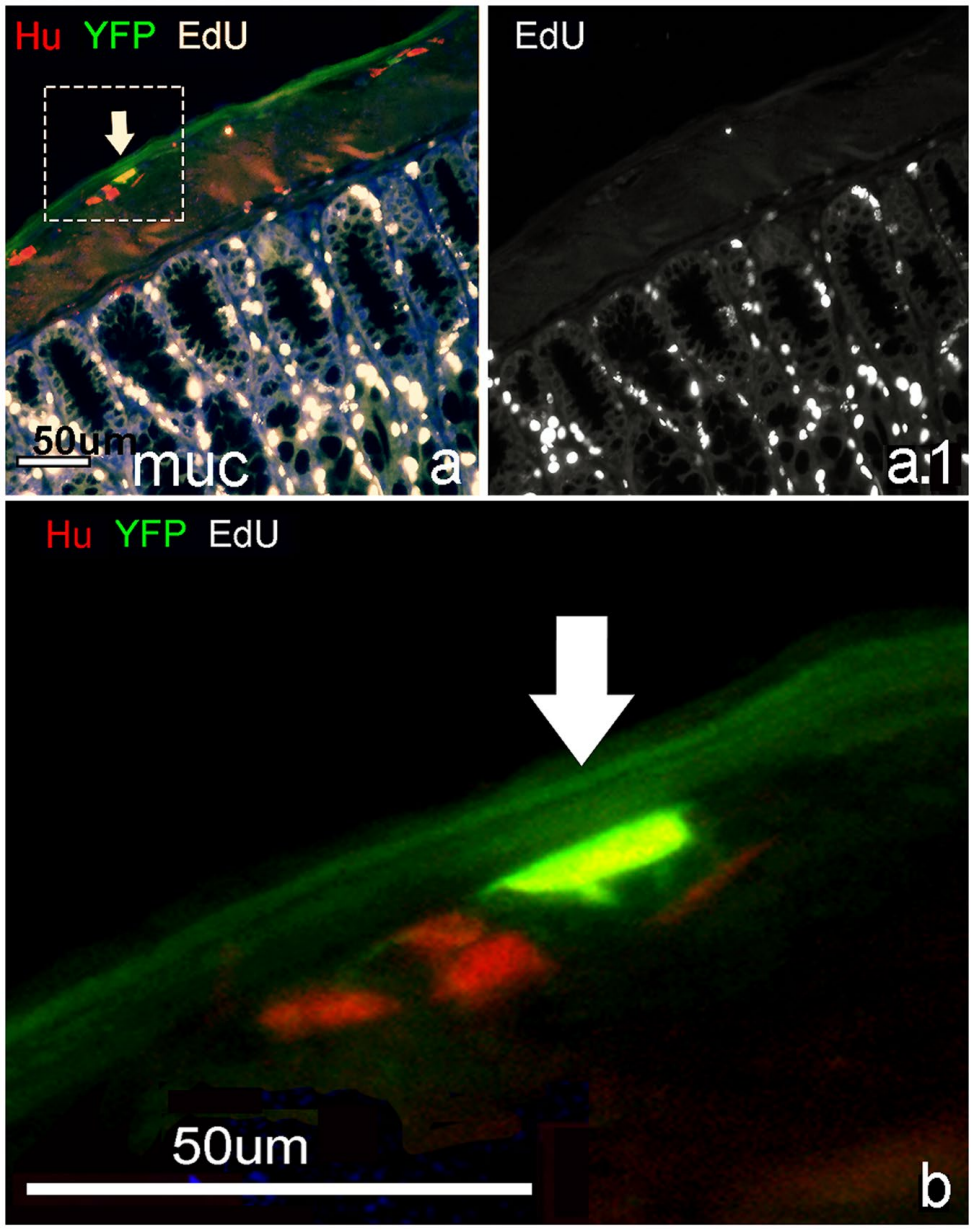
Glia give rise to neurons without new DNA synthesis. EdU was administered i.p. daily (50 mg/Kg) to Sox2creER:YFP mice receiving DSS for 7 days and examined 2 days later. EdU incorporation was seen in the mucosa (muc a, a.1), but not in Hu+ or Hu + YFP+ neurons (arrow). (**b**) Magnified view of myenteric ganglia. (Fig. 4 was taken at 200X, except closeup magnification 4.**b**).

Wholemount immunohistochemistry was performed on myenteric plexus preparations from the distal colon of 4 month-old Sox2CreER:YFP mice treated with DSS. EdU was given to Sox2CreER:YFP mice during DSS treatment to label dividing cells, which are readily seen in the mucosa (Fig. 4). However, no EdU labeling was identified in >10,000 Hu+ neurons counted, including those expressing YFP (Fig. 4, arrow). Therefore, despite the significant increase in YFP+ neurons induced by colitis (Fig. 3), no DNA replication was observed in these new enteric neurons (Fig. 4).

### Colitis causes PLP1-expressing cells to differentiate into enteric neurons

**Figure 5:**
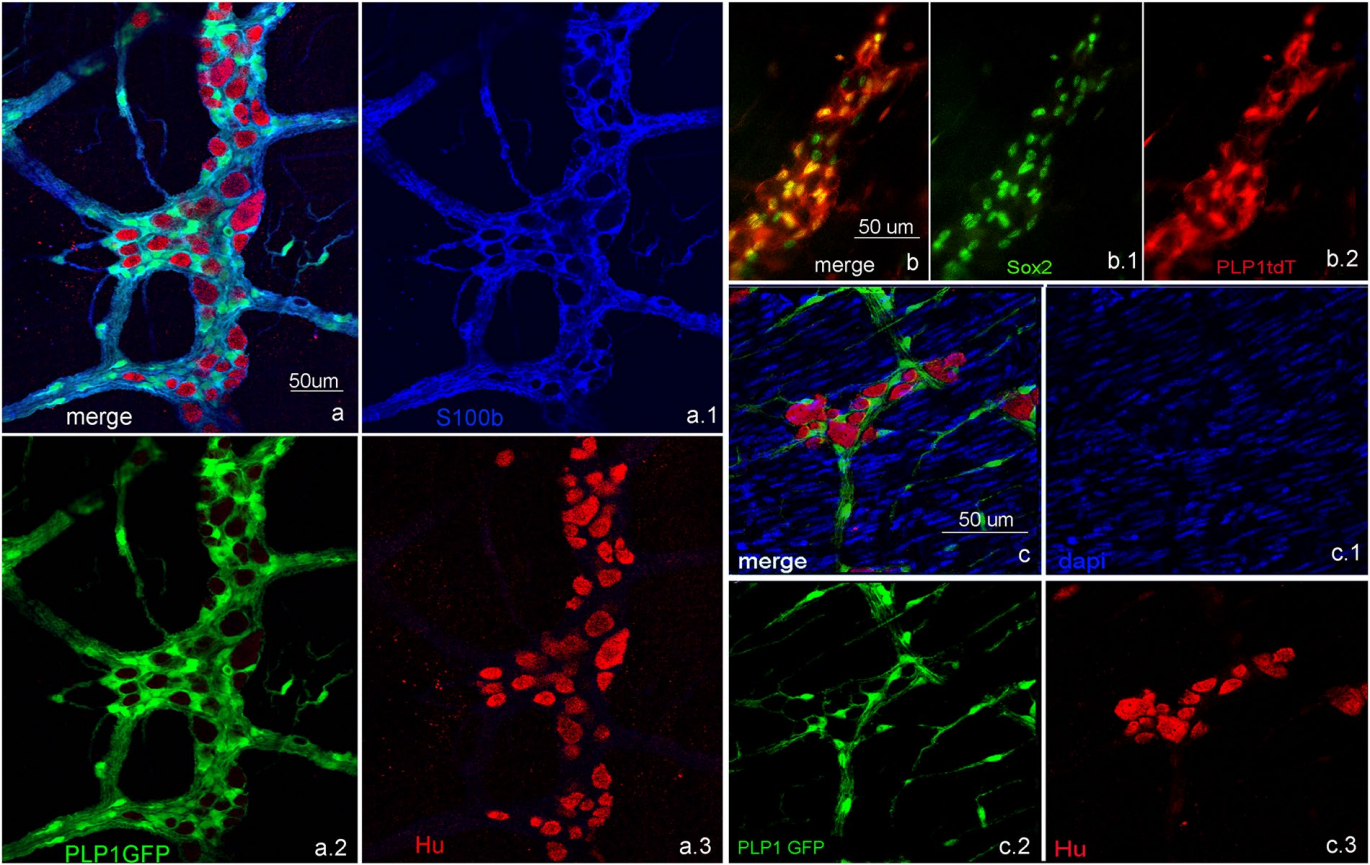
PLP1 is expressed by enteric glia and not neurons. PLP1tdT+ cells co-express S100b (**a**). PLP1tdT expressing cells also express Sox2 (**b**). In PLP1GFP mice, enteric neurons are not GFP+ in the adult gut (**c**). (All images of Fig. 5 were taken at 200X).

To further identity the immunophenotype of the newly born neurons, we repeated the cell-fate experiments using a second enteric glial cell marker, proteolipid protein-1 (PLP1)^16^. In PLP1CreER:tdT mice, enteric glia widely express tdT and most tdT+ glia co-express S100b (Fig. 5a). Extensive overlap between tdT+ cells is also seen with Sox2 (Fig. 5b). This expression pattern is consistent with what is observed in colon isolated from PLP1GFP mice, where enteric glia express PLP1 and enteric neurons do not (Fig. 5c). Similar to Sox2CreER:YFP mice, in PLP1creER:tdT mice not given tamoxifen no fluorescently labeled cells are seen in the colonic ENS.

**Figure 6:**
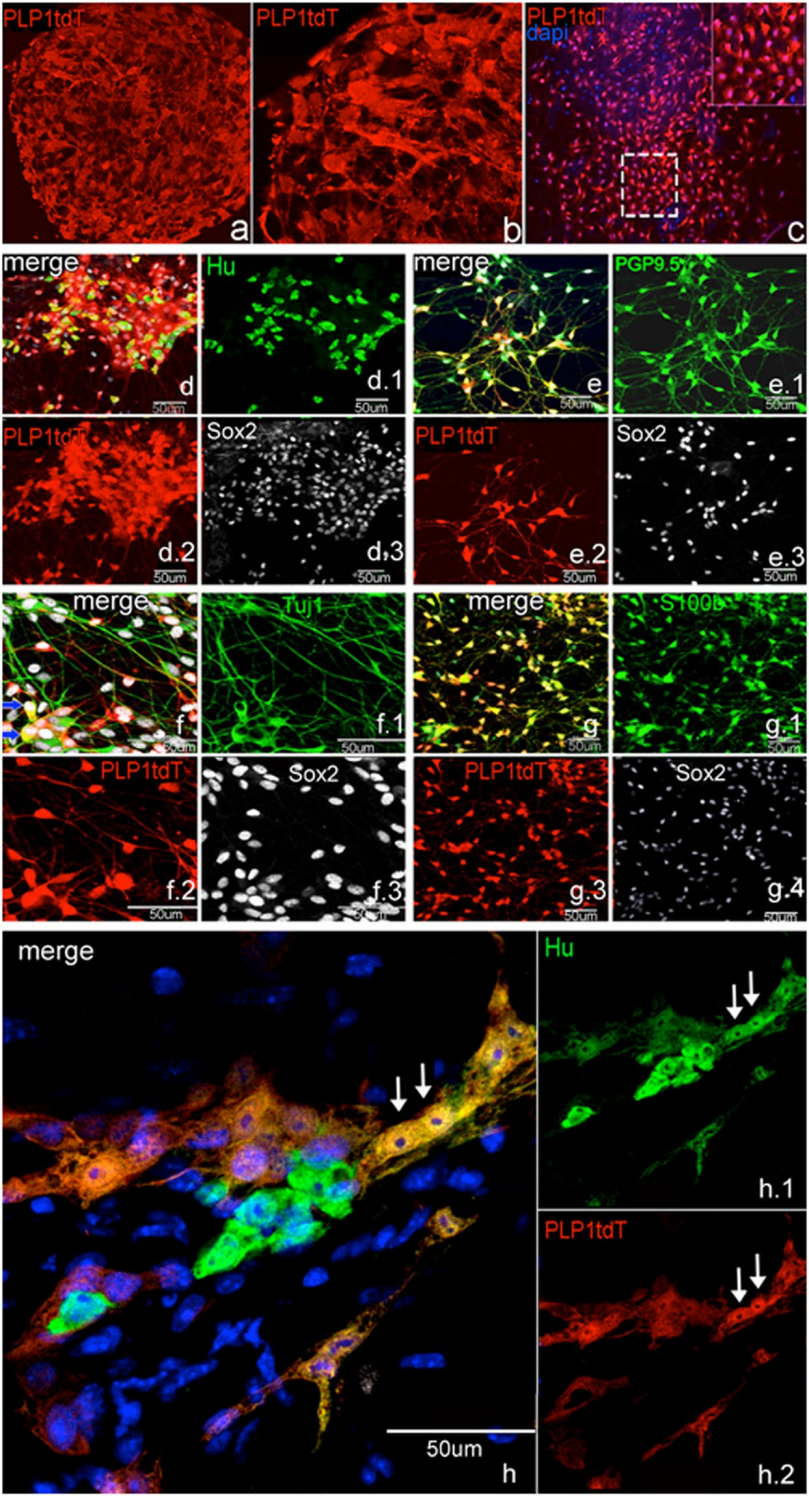
PLP1+ enteric glia give rise to neurons *in vitro* and *ex vivo*. Enteric neurospheres derived from PLP1CreER:tdT mice contain >90% tdT+ cells by confocal imaging (**a**,**b** close up of boxed area in **a**). The majority of cells dissociated from these neurospheres are tdT+ (**c**). Following culture, PLP1CreER:tdT-derived cells are positive for Hu and Sox2 (**d**), PGP9.5 and Sox2 (**e**), Tuj1 and Sox2, ((**f**) blue arrows denote triple-expressing cells), and S100b and Sox2 (**g**). Cultured colonic LMMP from PLP1CreER:tdT mice also shows tdT+/Hu+ double-expressing cells (**h**, arrows). All images of Fig. 6 were taken at 100X except closeup. (5**b** and **f**,**h** at 200x).

To test whether PLP1-tdT expressing cells can give rise to neurons *in vitro*, neurospheres were generated from 5 month-old PLP1CreER:tdT+ intestine. After 1 week in culture, neurospheres contained >90% tdT+ cells, quantified by confocal imaging as well as tissue dissociation and cell quantification of neurosphere-derived single cells (Fig. 6a–c). Neurospheres were then cultured in differentiation conditions for 7 days and immunostained with neuronal (Tuj1, Hu, PGP 9.5) and glial markers (S100b). Despite the fact that 87 ± 11% of tdT+ cells co-expressed S100b (Fig. 6g), there was a high rate of co-expression of tdT+ cells with each of the neuronal antibodies (Fig. 6d–f). Most tdT+ cells also expressed Sox2 (Fig. 6d–g). To further confirm these findings, colonic longitudinal muscle-myenteric plexus (LMMP) was prepared from 5 month-old adult PLP1CreER:tdT mice and cultured *ex vivo* for 3–6 days. Co-expression of PLP1tdT and Hu in the LMMP before dissociation was very rare, with only 0.3% of neurons expressing tdT. In contrast, after the culture period, 37 ± 5 % tdT+ cells expressed Hu (Fig. 6h).

**Figure 7:**
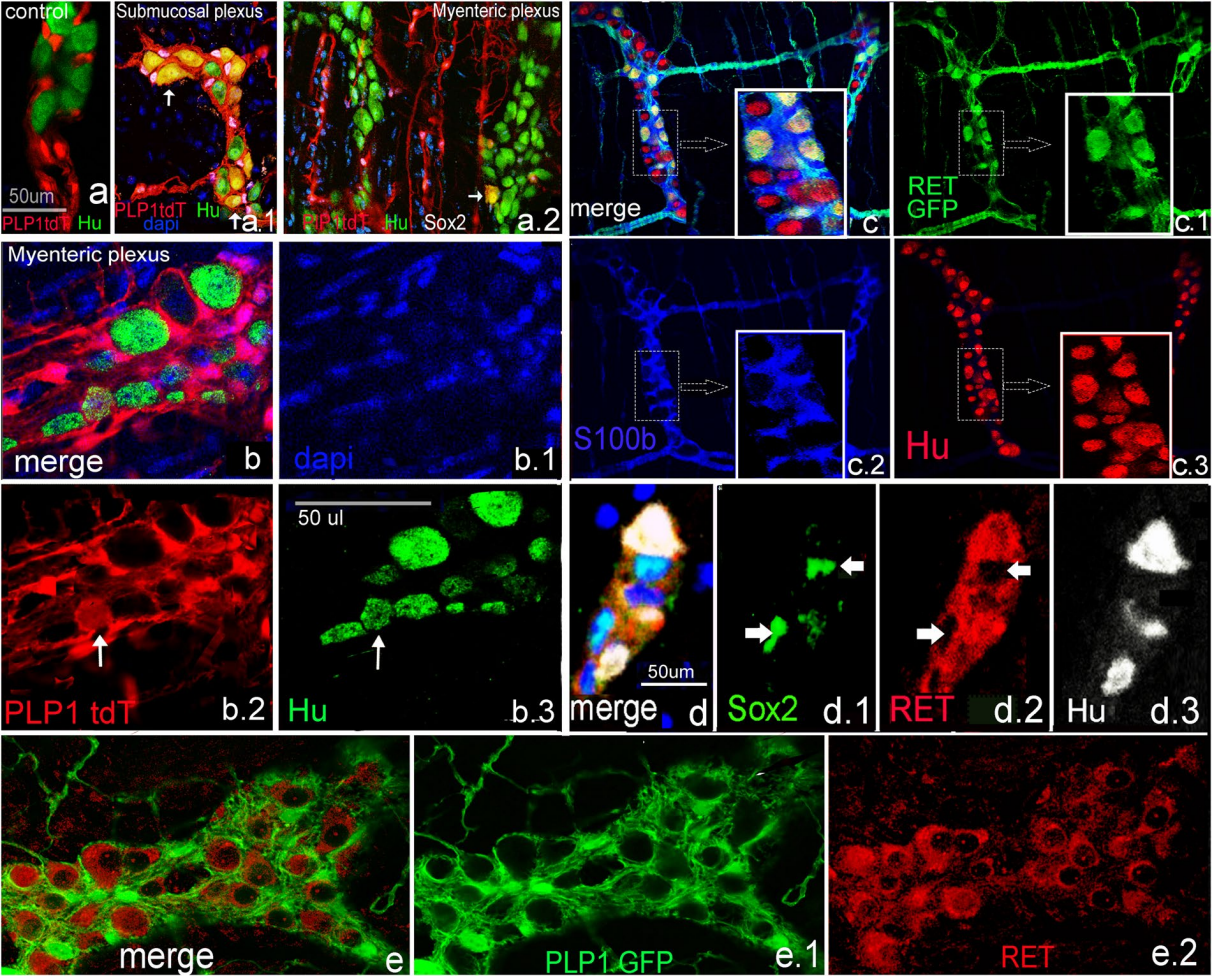
PLP1+ enteric glia give rise to neurons after colitis. PLP1CreER:tdT is not normally expressed in neurons in adult mice (**a**). However, mice give rise to Hu+ neurons following colitis in the submucosal (**a**.1) and myenteric plexus (**a.2**,**b–b.3**, arrows point to PLP1tdT + Hu+ cells). In normal, non-inflamed colon, glial cells do not express RET, which is normally expressed by neurons and progenitors (**c**–**c.3**). Similarly, Sox2+ cells do not co-express RET in the adult ENS (**d**, arrows), nor do PLP1+ cells (**e**–**e2**). (Images 7.**a**, and 7.**c** were taken at 200x except for closeups, 7.**b**–**e** were taken at 400X).

To determine whether PLP1-expressing cells are capable of undergoing neurogenesis *in vivo*, we subjected adult mice (>4 months old) to DSS colitis. tdT+ cells gave rise to neurons in both the submucosal and myenteric plexuses (Fig. 7a.1–a.2). We had previously verified that there were no TdT+ neurons in vehicle-treated mice (Fig. 7a). To distinguish whether the observed neurogenesis was due to glial transdifferentiation or to differentiation of neural progenitor cells, we investigated whether any Sox2+ or PLP1+ cells normally co-express RET, a protein expressed by both neurons and progenitors. RET is not expressed by S100+ mature glia (Fig. 7c). Similarly, we could not find Sox2+ cells that express RET in adult colon (Fig. 7d, arrows). In addition, PLP1GFP colon was analyzed by examining >1000 myenteric and submucosal ganglia and no GFP+ cells co-expressed RET (Fig. 7e).

### New neurons born in the setting of colitis downregulate Sox2 expression

**Figure 8:**
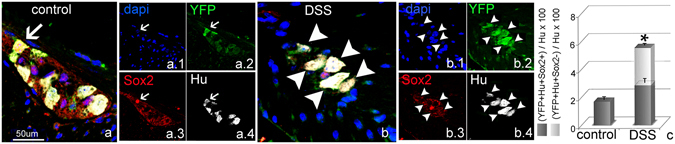
Colitis induces YFP + Hu+ cells that subsequently downregulate Sox2 expression. In the myenteric ganglia of control mice, all YFP + Hu+ cells (YFP green, Hu white) express Sox2 (red) (**a**–**a.4** arrow). Following DSS 50% of the YFP+ neurons no longer express Sox2 protein (**b**–**b**.**4** arrowheads). A 25% increase in Hu + YFP + Sox2+ cells is still observed in DSS-treated mice, (**c**, blue bar), but many of the new Hu+ neurons are negative for Sox2 protein expression (**c**, green bar). *p < 0.05. (All Images were taken at 200x).

The co-expression of Hu and Sox2 in newly born neurons was observed using Sox2CreER:YFP mice, in which YFP continues to be expressed even if Sox2 is downregulated. Since neuronal differentiation is typically associated with downregulation of Sox2^24^, we asked whether this was occurring in the ENS as well. Tamoxifen was administered for 3 consecutive days to adult Sox2CreER:YFP mice, followed by a 21-day chase. DSS was then orally administered for 7 days and mice sacrificed 3 days later. Sox2 immunohistochemistry was performed in order to determine whether Sox2 protein remained actively expressed in YFP+ cells. The number of YFP + Hu + Sox2+ and YFP + Hu + Sox2− cells was quantified. In controls, all YFP + Hu+ cells continued to express Sox2+ at the time of sacrifice (Fig. 8a,c). As described above, this represented only 1% of all Hu+ enteric neurons. In DSS-treated mice, a three- to four-fold increase in YFP + Hu+ cells was observed (Fig. 8c). While there was a small increase of 25% in Hu + YFP + Sox2+ cells (Fig. 8c), approximately half of YFP + Hu+ cells were negative for Sox2 expression at the time of sacrifice (Fig. 8b,c). These findings suggest that colitis induces the birth of new neurons, many of which subsequently downregulate Sox2.

### Human colitis is associated with enteric neurogenesis

**Figure 9:**
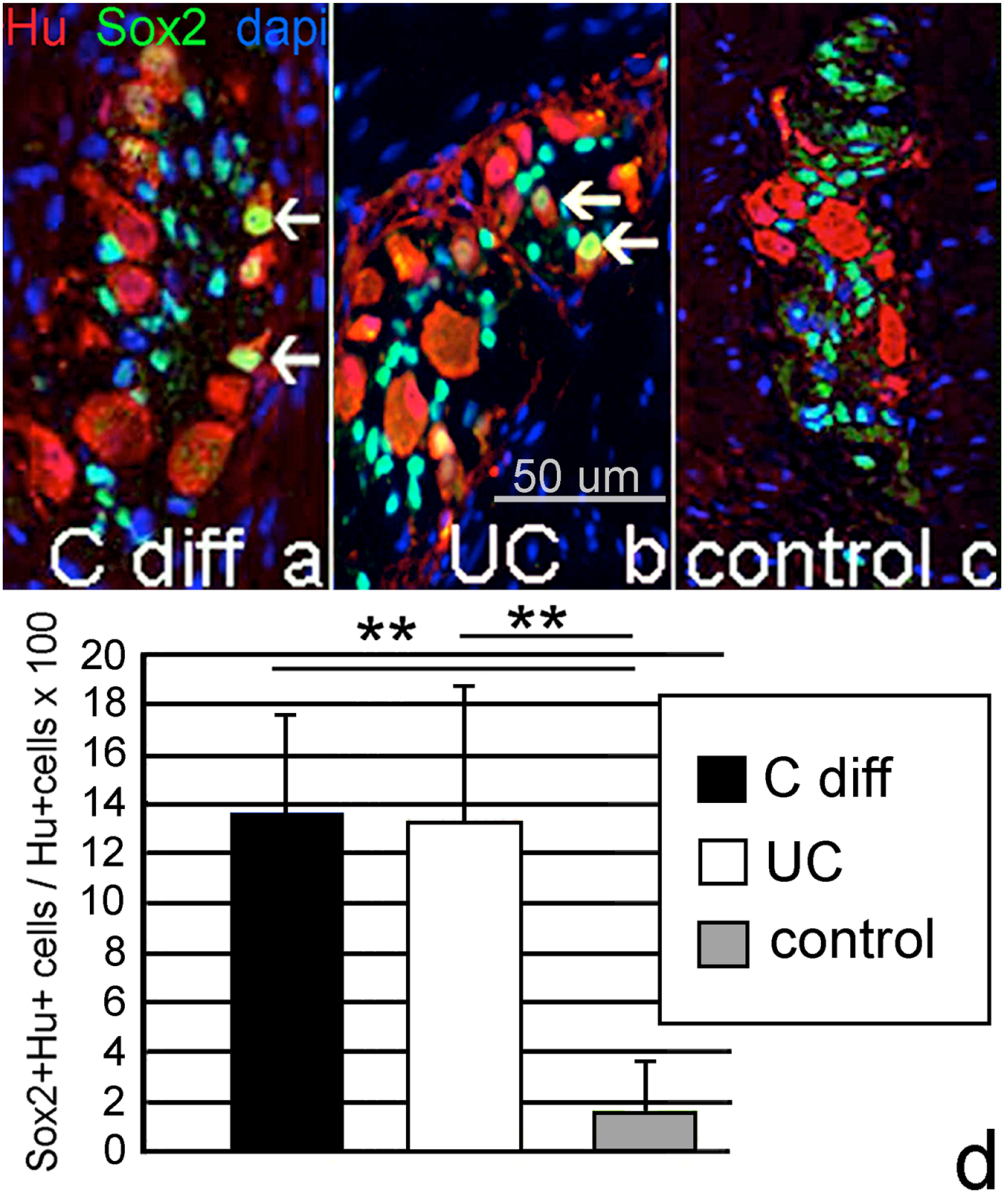
Infectious and inflammatory colitis in humans leads to an expansion of Sox2 + Hu+ neurons. Patients with *Clostridium difficile* (n = 15) (**a**) or ulcerative colitis (n = 15) (**b**) have significantly more Sox2 + Hu+ neurons than control colon (**c**), where Sox2 + Hu+ neurons are infrequently observed (**d**). (All Images were taken at 200x).

Determining whether neurogenesis occurs in human colitis is difficult given the challenge of accurately assessing neuronal numbers in clinical samples and the difficulty in obtaining samples at multiple time points. We therefore examined the presence of Sox2 + Hu+ enteric neurons in patients with inflammatory or infectious colitis as a proxy for inflammation-induced neurogenesis and also to determine whether a similar Sox2+ cell may give rise to neurons in inflamed human colon. Full-thickness biopsies were obtained from 15 adults with infectious (*Clostridium difficile*) colitis (average age 72.5 (range 44–92)) who had a colectomy due to their disease, 15 patients with ulcerative colitis (average age 35.1 (range 6–68), and 5 healthy adult controls (average age 73 (range 58–82)). Patients with colitis of either type had an average of 14% (range 7–19%) Hu+ neurons that co-expressed Sox2, while double-labeled cells were seen only in approximately 2% in control colon (Fig. 9a–d).

### Newly formed neurons predominately express calretinin and form synapses

**Figure 10:**
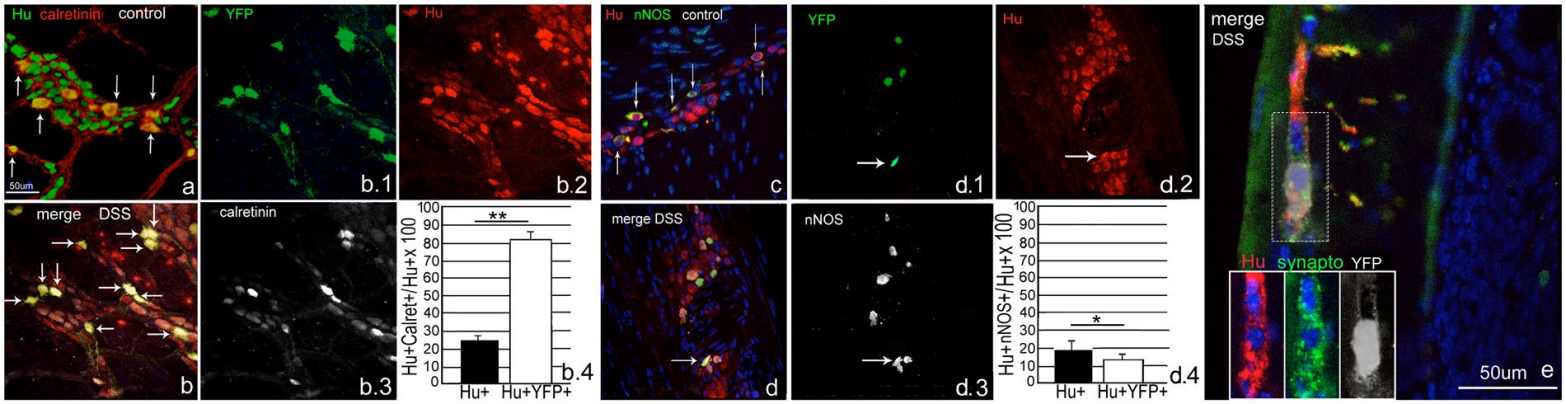
Newly formed neurons following colitis are predominantly calretinin excitatory motor neurons, which form synapses. In the myenteric ganglia of control mice, approximately 25% of Hu+ neurons express calretinin ((**a**) Hu green, calretinin red, arrows point to double-expressing cells). In our model, however 82% of YFP+ neurons (thus the newly formed neurons) express calretinin (**b**–**b.4** YFP green, calretinin white, Hu red. Arrows in b point to triple-expressing cells). nNOS is normally expressed by 19% of Hu+ neurons in control ((**c**) nNOS green, Hu red. Arrows point to double-expressing cells), but was only found to be expressed by 13% of YFP+ neurons post-colitis (**d.1**–**d.4** YFP green, Hu red and nNOS white. Arrows point to triple-expressing cells). The newly formed neurons synthesize synapses as manifested by synaptophysin expression (**e**). *p < 0.05, **p < 0.01. (All Images were taken at 200x).

To understand the expression profile of the newly generated neurons, we investigated the expression pattern of the motor excitatory neurotransmitter calretinin and the expression of the predominantly inhibitor neurotransmitter nitric oxide, represented by the expression of nNOS. We found that 82+/−5% of the newly formed YFP-expressing neurons were calretinin-expressing and thus excitatory. This is in contrast with only a 25+/−4% calretinin neuronal expression in control (p < 0.01) (Fig. 10a–b.4). Only 13+/−2% of YFP+ neurons expressed nNOS, which was significantly less than control (19+/−4%, p < 0.05) (Fig. 10c–c.5).

The new formed neurons were also found to express synpatophysin around their soma and neurites (Fig. 10d) in the distribution expected when neurons forms synaptic connections with the cells in their vicinity. These neurotransmitter and synaptophysin expression studies are very suggestive that the newly formed neurons function in a predominantly excitatory role.

## Discussion

Postnatal neurogenesis occurs in the central nervous system (CNS)^25^ and the mechanisms, triggers, and cell source of postnatal CNS neurogenesis are partially understood^26, 27^. In the CNS, glial cells are able to differentiate into neurons in response to injury^28–30^. These cells share many similarities with enteric glia, including expression of S100b and GFAP^31^, leading us to hypothesize that enteric glia may be similarly able to replace neurons following intestinal injury. As the ENS is subject to injury from toxins, inflammation, aging, and surgery, one would anticipate that postnatal neurogenesis is necessary in order to replace injured neurons. Constitutive postnatal neurogenesis occurs in rodents in the first few months of life^5^ but has been more elusive to demonstrate in older animals. Liu *et al*.^4^ were the first to demonstrate neurogenesis in the adult intestine, showing BrdU incorporation into Hu+ myenteric neurons in mice receiving a 5HT_4_ agonist. Laranjeira *et al*.^5^ reported the presence of enteric neurogenesis in adult mice following chemical denervation of the gut, showing that the newly generated neurons were derived from Sox10+ cells, a population that represents either mature enteric glia or neural crest-derived progenitor cells in the gut. We reported an increase of neurons following colitis, yet the source of those new neurons was unknown^7^. We speculate that this may be a regenerative response after colitis-associated neuronal cell death^32^. In contrast, Joseph *et al*.^6^ did not observe neurogenesis in adult mice during aging, pregnancy, dietary changes, hyperglycemia, exercise, inflammation, irradiation, chemical denervation, or glial ablation. Recently, Uesaka *et al*. has described evidence to suggest that Schwann cell precursors may migrate into the postnatal gut and give rise to new neurons^33^. Thus, the existing evidence clearly suggests that postnatal intestinal neurogenesis can occur, although the cells that give rise to those new neurons and the stimuli that activate this process remain unknown. The difficulty in consistently demonstrating adult neurogenesis *in vivo* suggests that it may occur only under specific scenarios, including certain injury models, specific ages, and localized regions of the intestinal tract. The goal of this study was to better characterize the cell source and mechanisms underlying postnatal neurogenesis in the injured adult gut.

Prior *in vivo* studies have attempted to identify enteric neurogenesis by looking for mitotically active cells that express neuronal markers, and many were unsuccessful in doing so^4, 6^. By using this approach, only those who have used exogenous 5HT4 agonists^4^ or by inflicting a BAC-denervation lesion^5, 34, 35^ have succeeded in describing enteric neurogenesis in the adult. We also were unable to find evidence of neurogenesis using EdU incorporation, which led us to hypothesize that the new neurons may arise from differentiation of existing cells rather than from the proliferation of a progenitor cell. In particular, we were surprised to see that the number of neurons rise rapidly (within days) when using mucosal-submucosal colitis models, whereas the mitotically active postnatal neural progenitors would appear weeks after either the 5HT4 agonist or the instillation of BAC^4, 5, 34, 35^. Cell fate can change in certain settings without the cell passing through a bipotent progenitor state, and this process represents direct lineage reprogramming or fate conversion^36^. This occurs in the retina after activation of the canonical Wnt signaling pathway^37–39^ and in the carotid body chemoreceptor following hypoxia^40^. Fate mapping using a *Wnt1-Cre* reporter line demonstrated that the carotid body contains neural crest-derived glia-like cells with sphere-forming capacity, which can produce TH-positive neurons and smooth muscle cells *in vitro*. Upon activation *in vivo*, these cells downregulate the glial marker GFAP, become nestin-positive, and contribute to *de novo* neurogenesis^29, 30, 40^. CNS glia, are thus capable of neurogenic differentiation, supporting that a similar process may be possible in the gut.

We previously found that addition of a 5HT_4_ agonist to cultured enteric neurospheres promoted neuronal and glial cell proliferation^41^. However, despite active glial mitosis, we noted that the number of glial cells did not increase, suggesting the possibility that a subset of glia may be changing fate. In this study, we find that colitis induced by either DSS of *Citrobacter rodentium* infection leads to increased neuronal density, but without any observable thymidine analog uptake by enteric neurons. The timing, as described starts much earlier than the previously described neurogenesis models. To determine whether these new neurons arise from enteric glial cells, we examined Sox2 expression, which we, and others^23^, find limited to mature glial cells in the adult intestine. DSS colitis in Sox2CreER:YFP mice led to a significant expansion of Hu + Sox2YFP+ neurons, supporting a glial contribution to the expanded neuronal pool. Importantly, the newly generated Hu + Sox2YFP+ neurons did not incorporate EdU, suggesting that the neurogenesis response, could represents a glial cell undergoing neuronal transdifferentiation. In the setting of colitis, we have previously observed and reported that about 5% of enteric glia incorporate EdU^41^. Our results support the notion that enteric glia give rise to neurons *in vivo*, but despite this we do not see EdU in the neurons. This observation is the basis of ongoing experiments. One possibility is that there are subpopulations of glial cells, some of which proliferate in response to inflammation while others undergo neuronal differentiation. Another possibility is asymmetric cell division, with only one daughter cell containing new DNA. A third possibility is that a neural progenitor that resides within or close to the ENS is stimulated to differentiate but in this early phase does so without DNA synthesis.

We chose to study Sox2 given its role in the CNS, where it has been shown to be necessary and sufficient to reprogram astrocytes into neuroblasts in the adult mouse brain following injury^12^. Sox2 then may not only be a marker of glial-to-neuronal conversion, but may also play a role in the transdifferentiation process. If neural progenitors were giving rise to new enteric neurons in response to injury, one would expect DNA proliferation to occur in order for the progenitors to maintain a population of undifferentiated cells, unless local neural progenitors differentiate to neurons with colitis but do so, at least in this early phase, without undergoing DNA synthesis.

Neural progenitors in the gut positive for both Sox2+ and RET+ have been described at birth^42^. We and other have found a small number of cells in the adult ENS that co-express glial and neuronal markers, such as Hu and CD49b, Hu and Nestin, Hu and FoxD3 and Hu and Sox2^7, 43^. However, in adult mouse intestine we could find no cells that co-expressed Sox2 and RET, suggesting that if there is a pool of Sox2+ progenitors in the adult gut, it is likely a small population of cells. Because ant-RET antibodies may lack specificity or sensitivity, we also used the RET:GFP mouse to search for Sox2+ and RET+ progenitor cells, but were unable to find any double expressing glial cells.

**Figure 11:**
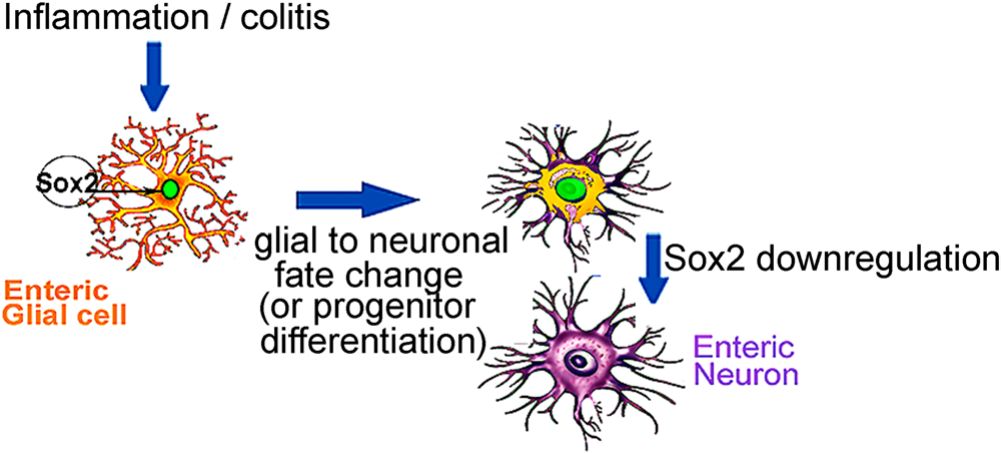
Inflammation induced early neurogenesis. In the present work, we show that inflammation can cause a rapid glial-to-neuronal fate change or neural progenitor differentiation, which can be identified by an expanding neuronal mass and a new population of Sox2 + Hu+ neurons. We hypothesize that these then downregulate Sox2 as they become mature neurons.

To further support our hypothesis of colitis-induced glial-derived neurogenesis *in vivo*, we tested our results in a second mouse model in which the fate of cells expressing the enteric glial marker, PLP1^16^, was followed after induction of colitis. As with Sox2, we found no RET expression in PLP1TdT+ enteric glia, unless the PLP1tdT+ cell also expressed the neuronal marker Hu and therefore represent newly born neurons. Thus we believe that the expression profile of the cells with neurogenic potential (S100b+, PLP1+, Sox2+, Ret−) is consistent with enteric glia and less likely to be progenitors. The extent of the injury-induced expansion of the neuronal population in the absence of observable mitosis further supports that lineage reprogramming of glial cells underscores this process. Collectively, these data support the notion that adult enteric neurogenesis is triggered by inflammation and identify enteric glia as the neurogenic cell source (Fig. 11). Further studies are necessary to determine whether a specific subpopulation of glia or progenitors is responsible for this homeostatic function and to define the precise signals that activate this process.

Our observations in human colitis suggest that a similar phenomenon of glial-to-neuronal fate conversion may occur in human enteropathies. We identified a dramatic seven-fold increase in the number of Hu + Sox2+ double-labeled enteric neurons in the patients examined, including individuals up to 82 years of age. The presence of these double-labeled cells suggests that colonic inflammation in humans may cause a subpopulation of Sox2-immunoreactive glial cells to switch to a neuronal fate. Another possible explanation is that a subset of neurons may begin to express Sox2 in humans with colitis. Definitive *in vivo* evidence of this will be difficult to obtain in humans since, unlike CreER-labeled tissue in mice, we cannot identify a cell that has turned off Sox2 expression. In the mice, we found that about half of the Sox2CreER:YFP+ neurons lose Sox2 expression, but the other half retain it. The proportion of Hu-expressing glial cells that retain Sox2 is still greater than baseline. In humans, we used antibodies to detect the presence of protein expression. We hypothesize that the increase in Sox2+ neurons in the samples represents only a fraction of glial cells that have undergone neuronal change-of-fate. Finally, we sought to find evidence of function by the newly generated neurons. We found that they predominantly express the excitatory neurotransmitter calretinin and that they form synapses, providing evidence that they are functioning predominantly as excitatory motor neurons. This calretinin-predominant pattern of expression was similarly found by Uesaka *et al*.^33^ in the neurons they found to be generated from Schwann cell progenitors, suggesting either a common pathway or perhaps that the molecular signaling from the injured colon lead to an increase in excitatory neural differentiation.

In conclusion, we present *in vitro* and *in vivo* evidence using transgenic mice models and immunofluorescence-based expression studies that demonstrate that colitis induces early enteric neurogenesis in adult mice and possibly in humans as well. The newly born neurons appear shortly after induction of inflammation, do not retain DNA label, and are derived from Sox2+ and PLP1 glial cells. They are predominantly calretinin expressing, and thus excitatory motor neurons. This may represent a different mechanism for adult enteric neurogenesis than that described in previous BAC-denervation and 5HT4 agonist models, as in these, the new neurons appear much later and a significant percentage of them display thymidine analog retention. These studies may advance our understanding of the effects of gut injury on the enteric nervous system and will provide insights into understanding the signals that control neurogenesis in the healthy and diseased intestine.

## References

[1] Srinivasan, S. & Wiley, J. W. New insights into neural injury, repair, and adaptation in visceral afferents and the enteric nervous system. *Curr Opin Gastroenterol* **16**, 78–82, doi:00001574-200001000-00014 [pii] (2000). 10.1097/00001574-200001000-00014 17024021

[2] Becker, L., Peterson, J., Kulkarni, S. & Pasricha, P. J. Ex vivo neurogenesis within enteric ganglia occurs in a PTEN dependent manner. *PLoS One* **8**, e59452, doi:10.1371/journal.pone.0059452 PONE-D-12-30691 [pii] (2013). 10.1371/journal.pone.0059452 PMC3602370 23527198

[3] Urban N, Guillemot F (2014). Neurogenesis in the embryonic and adult brain: same regulators, different roles. Front Cell Neurosci.

[4] Liu MT, Kuan YH, Wang J, Hen R, Gershon MD (2009). 5-HT4 receptor-mediated neuroprotection and neurogenesis in the enteric nervous system of adult mice. J Neurosci.

[5] Laranjeira C (2011). Glial cells in the mouse enteric nervous system can undergo neurogenesis in response to injury. J Clin Invest.

[6] Joseph, N. M. *et al*. Enteric glia are multipotent in culture but primarily form glia in the adult rodent gut. *J Clin Invest* **121**, 3398–3411, doi:10.1172/JCI5818658186 [pii] (2011). 10.1172/JCI58186 PMC3163971 21865643

[7] Belkind-Gerson J (2015). Colitis Induces Enteric Neurogenesis Through a 5-HT4-dependent Mechanism. Inflamm Bowel Dis.

[8] Coelho-Aguiar Jde M (2015). The enteric glia: identity and functions. Glia.

[9] Robins SC (2013). Extensive regenerative plasticity among adult NG2-glia populations is exclusively based on self-renewal. Glia.

[10] Heinrich C (2010). Directing astroglia from the cerebral cortex into subtype specific functional neurons. PLoS biology.

[11] Torper O (2013). Generation of induced neurons via direct conversion *in vivo*. Proc Natl Acad Sci USA.

[12] Niu W (2013). *In vivo* reprogramming of astrocytes to neuroblasts in the adult brain. Nat Cell Biol.

[13] Arnold K (2011). Sox2(+) adult stem and progenitor cells are important for tissue regeneration and survival of mice. Cell Stem Cell.

[14] Mallon BS, Macklin WB (2002). Overexpression of the 3′-untranslated region of myelin proteolipid protein mRNA leads to reduced expression of endogenous proteolipid mRNA. Neurochem Res.

[15] Doerflinger NH, Macklin WB, Popko B (2003). Inducible site-specific recombination in myelinating cells. Genesis.

[16] Rao, M. et al. Enteric glia express proteolipid protein 1 and are a transcriptionally unique population of glia in the mammalian nervous system. *Glia*, doi:10.1002/glia.22876 (2015). 10.1002/glia.22876 PMC4695324 26119414

[17] Chen CC, Louie S, McCormick B, Walker WA, Shi HN (2005). Concurrent infection with an intestinal helminth parasite impairs host resistance to enteric Citrobacter rodentium and enhances Citrobacter-induced colitis in mice. Infection and immunity.

[18] Mizoguchi, E. Chitinase 3-like-1 exacerbates intestinal inflammation by enhancing bacterial adhesion and invasion in colonic epithelial cells. *Gastroenterology* **130**, 398–411, doi:S0016-5085(05)02436-4 [pii]10.1053/j.gastro.2005.12.007 (2006). 10.1053/j.gastro.2005.12.007 16472595

[19] Belkind-Gerson J (2013). Nestin-expressing cells in the gut give rise to enteric neurons and glial cells. Neurogastroenterol Motil.

[20] Hotta R, Anderson RB, Kobayashi K, Newgreen DF, Young HM (2010). Effects of tissue age, presence of neurones and endothelin-3 on the ability of enteric neurone precursors to colonize recipient gut: implications for cell-based therapies. Neurogastroenterol Motil.

[21] Su CW (2012). Helminth infection impairs autophagy-mediated killing of bacterial enteropathogens by macrophages. J Immunol.

[22] Luperchio SA (2000). Citrobacter rodentium, the causative agent of transmissible murine colonic hyperplasia, exhibits clonality: synonymy of C. rodentium and mouse-pathogenic Escherichia coli. Journal of clinical microbiology.

[23] Heanue TA, Pachnis V (2011). Prospective identification and isolation of enteric nervous system progenitors using Sox2. Stem Cells.

[24] Graham V, Khudyakov J, Ellis P, Pevny L (2003). SOX2 functions to maintain neural progenitor identity. Neuron.

[25] Alvarez-Buylla A, Theelen M, Nottebohm F (1988). Mapping of radial glia and of a new cell type in adult canary brain. J Neurosci.

[26] Van Schepdael A, Ashbourn JM, Beard R, Miller JJ, Geris L (2013). Mechanisms of cell migration in the adult brain: modelling subventricular neurogenesis. Computer methods in biomechanics and biomedical engineering.

[27] Zhang RL, Zhang ZG, Chopp M (2005). Neurogenesis in the adult ischemic brain: generation, migration, survival, and restorative therapy. The Neuroscientist: a review journal bringing neurobiology, neurology and psychiatry.

[28] Heinrich C (2014). Sox2-mediated conversion of NG2 glia into induced neurons in the injured adult cerebral cortex. Stem Cell Reports.

[29] Guo Z (2014). In vivo direct reprogramming of reactive glial cells into functional neurons after brain injury and in an Alzheimer's disease model. Cell Stem Cell.

[30] Duan CL (2015). Striatal astrocytes transdifferentiate into functional mature neurons following ischemic brain injury. Glia.

[31] Burns AJ, Thapar N (2014). Neural stem cell therapies for enteric nervous system disorders. Nat Rev Gastroenterol Hepatol.

[32] Brown IA, McClain JL, Watson RE, Patel BA, Gulbransen BD (2016). Enteric glia mediate neuron death in colitis through purinergic pathways that require connexin-43 and nitric oxide. Cell Mol Gastroenterol Hepatol.

[33] Uesaka T, Nagashimada M, Enomoto H (2015). Neuronal Differentiation in Schwann Cell Lineage Underlies Postnatal Neurogenesis in the Enteric Nervous System. J Neurosci.

[34] Hanani M (2003). Regeneration of myenteric plexus in the mouse colon after experimental denervation with benzalkonium chloride. J Comp Neurol.

[35] Tamada H, Kiyama H (2016). Suppression of c-Kit signaling induces adult neurogenesis in the mouse intestine after myenteric plexus ablation with benzalkonium chloride. Sci Rep.

[36] Ninkovic J, Gotz M (2015). How to make neurons–thoughts on the molecular logic of neurogenesis in the central nervous system. Cell Tissue Res.

[37] Liu H (2007). Ciliary margin transdifferentiation from neural retina is controlled by canonical Wnt signaling. Dev Biol.

[38] Opas M, Davies JR, Zhou Y, Dziak E (2001). Formation of retinal pigment epithelium in vitro by transdifferentiation of neural retina cells. The International journal of developmental biology.

[39] Opas M, Dziak E (1998). Direct transdifferentiation in the vertebrate retina. The International journal of developmental biology.

[40] Pardal R, Ortega-Saenz P, Duran R, Lopez-Barneo J (2007). Glia-like stem cells sustain physiologic neurogenesis in the adult mammalian carotid body. Cell.

[41] Belkind-Gerson J (2015). Colitis induces enteric neurogenesis through a 5-HT4-dependent mechanism. Inflamm Bowel Dis.

[42] Vohra BP (2006). Differential gene expression and functional analysis implicate novel mechanisms in enteric nervous system precursor migration and neuritogenesis. Dev Biol.

[43] Musser MA, Correa H, Southard-Smith EM (2015). Enteric neuron imbalance and proximal dysmotility in ganglionated intestine of the Sox10Dom/+ Hirschsprung mouse model. Cell Mol Gastroenterol Hepatol.

